# The origin of the skewed amplitude distribution of spontaneous excitatory junction potentials in poorly coupled smooth muscle cells

**DOI:** 10.1016/j.neuroscience.2006.11.054

**Published:** 2007-03-02

**Authors:** J.S. Young, K.L. Brain, T.C. Cunnane

**Affiliations:** Department of Pharmacology, University of Oxford, Mansfield Road, Oxford OX1 3QT, UK

**Keywords:** calcium imaging, confocal microscopy, neurotransmission, smooth muscle, ATP, sEJP, α-SNAP, α–soluble *N*-ethylmaleimide-sensitive factor attachment protein, BAPTA-1 AM, 1,2-bis(*o*-aminophenoxy)ethane-*N*,*N*,*N*′,*N*′-tetraacetic acid–1 acetoxymethyl ester, EJP, excitatory junction potential, LTX, α-latrotoxin, NCT, neuroeffector calcium transient, NF449, 4,4′,4″,4‴-[carbonylbis[imino-5,1,3-benzenetriyl bis(carbonyl-imino)]]tetrakis(benzene-1,3-disulfonic acid), PSS, physiological salt solution, sEJP, spontaneous excitatory junction potential, SMC, smooth muscle cell

## Abstract

The skewed amplitude distribution of spontaneous excitatory junction potentials (sEJPs) in the mouse vas deferens and other electrically-coupled smooth muscle syncytia has been attributed to electrically-attenuated depolarizations resulting from the spontaneous release of quantized packets of ATP acting on remote smooth muscle cells (SMCs). However, in the present investigation surface SMCs of the mouse isolated vas deferens were poorly electrically coupled, with input resistances (176±18 MΩ, range: 141–221 MΩ, *n*=4) similar to those of dissociated cells. Furthermore, the amplitude of evoked EJPs was more variable in surface compared with deeper SMCs (*F* test, *F*=17.4, *P*<0.0001). Using simultaneous electrophysiology and confocal microscopy to investigate these poorly-coupled cells, it is shown that α-latrotoxin-stimulated sEJPs correlate, in timing (median delay ranged from −30 to −57 ms, *P*<0.05 in all experiments, *n*=5) and amplitude (Pearson product moment correlation, ρ>0.55 and *P*<0.001), with purinergic neuroeffector Ca^2+^ transients (NCTs) in SMCs. The temporal correlation between sEJPs of widely ranging amplitude with NCTs in the impaled SMC demonstrates that all sEJPs could arise from neurotransmitter action on the impaled cell and that the skewed distribution of sEJPs can be explained by the variable effect of packets of ATP on a single SMC. The amplitude correlation of sEJPs and NCTs argues against the attenuation of electrical signal amplitude along the length of a single SMC. The skewed sEJP amplitude distribution arising from neurotransmitter release on single SMCs is consistent with a broad neurotransmitter packet size distribution at sympathetic neuroeffector junctions.

The quantal basis of neurotransmission at the skeletal neuromuscular junction was identified by del Castillo and Katz (1954), in their reanalysis of experiments by Fatt and Katz (1952). They concluded that “transmission at a nerve-muscle junction takes place in all-or-none ‘quanta’ whose sizes are indicated by the spontaneously occurring miniature (end-plate potential) discharges.” The narrow, Gaussian amplitude distribution of the miniature end-plate potentials (their Fig. 7), the invariance of this distribution as the Ca^2+^ concentration changed and the existence of a multimodal histogram of excitatory postjunctional potentials imply that evoked neurotransmitter release causes integer multiples of a consistent unitary electrical response. The distribution of packet sizes released from sympathetic postganglionic terminals has been much harder to study because the electrical syncytium that arises through the coupling of smooth muscle cells (SMCs) makes it difficult to distinguish local from distant neurotransmitter release sites during intracellular recording. To tackle this problem, several different approaches have been tried. By differentiating the excitatory junction potential (EJP) it is possible to identify intermittent ‘discrete events’ buried within the depolarization phase (Blakeley and Cunnane, 1979), which indicate the intermittent release of neurotransmitter packets. Some of these discrete events had sizes comparable to the amplitude of the derivative of spontaneous excitatory junction potentials (sEJPs), which argues that the same types of packets contribute to both events, but does not indicate that evoked release is an integral multiple of unitary packets. A further approach is to measure local extracellular potential changes arising following both spontaneous and evoked neurotransmitter release: the excitatory junction currents (sEJCs and EJCs; Brock and Cunnane, 1987). This approach demonstrates intermittent neurotransmitter release with a broad packet size. A development of this approach to study visualized varicosities under conditions of low release probability with small electrode tips (Macleod et al., 1994) demonstrated that, at 1 mM Ca^2+^, the truncated amplitude distribution of EJPs was broad (their Fig. 3B), arguing against packets of uniform size; that packet size was not uniform was implicit in the model they used to fit the results at higher external Ca^2+^ concentrations, where the basic packet size varied as a gamma variate (Robinson, 1976). Further evidence for a broad amplitude distribution of the fundamental packet size at the autonomic neuroeffector junction has come from the use of laser-scanning confocal microscopy to image purinergic neuroeffector Ca^2+^ transients (NCTs) on an impulse-to-impulse basis at individual neuroeffector junctions (Brain et al., 2002, 2003). NCTs arise when intermittently-released packets of ATP activate local P2X_1_ receptors, causing a local increase in smooth muscle Ca^2+^ concentration. The finding that these local Ca^2+^ transients are amplified by Ca^2+^-induced Ca^2+^ release in the SMC, however, means that the broad amplitude distribution of NCTs at a single junction cannot be used to imply a broad distribution of basic packets size (Brain et al., 2003).

The mouse vas deferens, unlike that of the guinea pig, provides a good model to study quantal neurotransmission because there is already good evidence that the SMCs are poorly electrically coupled; in particular, it has not been possible to detect electronic potentials spreading from cell-to-cell or across the mouse vas deferens (Holman et al., 1977).

By combining confocal imaging with simultaneous intracellular electrophysiological recording in the mouse isolated vas deferens, it is possible to establish whether a correlation occurs between a traditional electrophysiological approach to monitoring neurotransmitter release, the EJP, and recently-developed optical approaches. Moreover, the combined techniques permit a study of the effective neurotransmitter packet size at the autonomic neuroeffector junction.

## Experimental procedures

### Ca^2+^ indicator loading

Eight- to 12-week-old Balb/c mice (Harlan, Bicester, Oxfordshire, UK) were killed by cervical fracture and both vasa deferentia removed. Efforts were made to minimize the number of animals used and their suffering; all experiments were carried out in accordance with the UK Animals (Scientific Procedures) Act 1986 and European Communities Council Directive 86/09/EEC. The connective tissue around each vas deferens was carefully dissected in order to obtain clear images of SMCs and to remove any ganglia located close to the prostatic end.

Each vas deferens was then exposed to 10 μM Oregon Green 488 1,2-bis(*o*-aminophenoxy)ethane-*N*,*N*,*N*′,*N*′-tetraacetic acid–1 acetoxymethyl ester (BAPTA-1 AM) (Invitrogen, Paisley, Renfrewshire, UK) in 1% dimethyl sulfoxide/0.2% pluronic F-127 (Sigma-Aldrich, St. Louis, MO, USA) in physiological salt solution (PSS) for 2 h at 36 °C. Each tissue was then cut longitudinally to create a flat sheet and rinsed in PSS, bubbled with 95% O_2_/5% CO_2_, for at least 10 min. Tissues were pinned flat, serosal side up in a Sylgard-lined organ bath, and mounted on the stage of an upright confocal microscope. The PSS contained (mM): NaCl 118.4, NaHCO_3_ 25.0, NaH_2_PO_4_ 1.13, KCl 4.7, CaCl_2_ 1.8, MgCl_2_ 1.3 and glucose 11.1. The pH was maintained at 7.4 and the solution oxygenated by continuous bubbling with 95% O_2_/5% CO_2_.

### Confocal microscopy

The vas deferens was placed in a chamber that was continuously superfused with standard PSS (bath temperature 33–34 °C). Images were acquired with a Leica SP2 upright confocal microscope (Leica Microsystems, Milton Keynes, UK). A series of 100 or 200 frames was captured at approximately 5 or 13.5 Hz to generate one image set. Such sets were acquired once every minute. Between 8 and 12 such sets were acquired for each SMC.

Surface SMCs do not lie perfectly orthogonal to the optical axis of the microscope, and therefore measurement of SMC lengths required this finding to be taken into consideration. When measuring cell lengths, a series of high-resolution (1024×1024 pixels) images was taken at intervals along the *z* axis. An average was then taken of the resultant series (‘z-stack’) of images, and the *x*–*y* projected length was calculated using a polygon tool function of Canvas (version 9, ACD Systems, Miami, FL, USA). The *z*-component was calculated by measuring the focal plane position at each end of the cell, so that a more precise length in three-dimensional space could be calculated using Pythagoras’ Theorem.

### Image analysis

Image analysis was performed with the stack profile function of the Leica LCS software or using an Image J (http://rsb.info.nih.gov/ij/download.html) plug-in written by R. J. Amos. In the first frame of the image series a region of interest was established which encompassed the portion of a SMC visible within the confocal plane. The fluorescent signal in this region was measured over time throughout the image set. Data were exported to Excel (Microsoft, Redmond, WA, USA) for formatting and then to Spike 2 (Cambridge Electronic Design, Cambridge, UK) for analysis in conjunction with electrophysiological recordings.

### Electrophysiology

Conventional intracellular recording techniques were used to record sEJPs in SMCs (see Brock and Cunnane, 1992). Each vas deferens was superfused with PSS and drugs were applied by swapping the perfusion solution to one containing the drug at the required final bath concentration. Preparations were perfused with prazosin (100 nM; Sigma-Aldrich) and α-latrotoxin (LTX) (25 pM; Sigma-Aldrich) for 30 min prior to recordings. The low concentration of latrotoxin was used to induce a mildly elevated rate of spontaneous neurotransmitter release so that a sufficient number of events could be detected within a fixed imaging period, but not so high that the majority of events could not be independently measured. Microelectrodes (tip resistances of 140–160 MΩ) were filled with Texas Red (2 mM filtered in 5 M potassium acetate; weight 625 Da; Invitrogen). The membrane potential was measured with an Axoclamp 2B (Axon Instruments, Sunnyvale, CA, USA) in bridge mode, in conjunction with a frame-coupled TTL output from the microscope to allow temporal correlation of electrophysiological and confocal recordings.

Recordings of electrically-evoked EJPs were achieved by applying rectangular pulses (0.6 ms duration; voltage amplitude at twice the threshold for eliciting EJPs, typically around 20 V) delivered through Ag/AgCl electrodes positioned around the prostatic end of the vas deferens. In some experiments, ‘non-surface’ SMCs were impaled using an almost vertical electrode approach (in the absence of confocal microscopy) with no attention paid to remaining close to the serosal surface.

In additional experiments, surface SMCs were voltage-clamped at various values of alternating depolarization and hyperpolarization with respect to their resting membrane potential (mean −65.1±2 mV) for 200 ms, with approximately 10 s between steps. The input resistance was calculated for the linear part of the voltage–current relationship (around the resting membrane potential). Both inward and outward currents were identified during depolarizing steps (in two of four cells), and in one cell, an inward current was observed following a hyperpolarizing step (data not shown). These phenomena were not further investigated as part of the present study.

The voltage and current were digitized (at 5 kHz and 1 kHz, respectively) with a PowerLab system (ADInstruments, Chalgrove, UK). Recordings of sEJPs were exported from Chart 4.2 (ADInstruments) for analysis with Spike 2. The amplitudes of EJPs were calculated using Chart 4.2 (ADInstruments).

The microelectrode was always within the field of view of the confocal image. On completion of each experiment, the SMC recorded from was labeled by injecting Texas Red by iontophoresis (current injection, +2 nA, 15 s).

### Analysis of correlations between NCTs and sEJPs

Analysis of the amplitudes of sEJPs and NCTs was performed on data where confocal images were acquired at 5 Hz. To obtain a more accurate measurement of the temporal relationship between sEJPs and NCTs, a faster acquisition rate of approximately 13.5 Hz (74 ms frame duration) was used.

The peak amplitudes and timings of both sEJPs and NCTs were calculated using a custom-written script for Spike 2. The occurrence of sEJPs was defined by their amplitude (≥2 mV). The threshold for determining NCTs varied between recordings from different SMCs. The threshold was adjusted until it provided a specificity comparable to manual counting (ΔF/F range of 0.05–0.1).

To investigate the temporal correlation between changes in the fluorescent signal and sEJPs, the time from each change in fluorescent signal to the nearest sEJP was measured (referred to as delay). To determine whether these events displayed a significant temporal correlation, the distribution of delays was compared (using chi-square tests) with the expected distribution under the assumption that sEJPs and NCTs were uncorrelated. The expected distribution of delays is the double exponential or Laplace distribution,

(1) $$P=\nu e^{-2\nu \left|t\right|}$$

where ν is the frequency of sEJPs. To maximize the power of the chi-square analysis the data were binned so that the expected number of events was constant in all bins. The *n*th positive boundary to give a relative bin size of ‘*d*’ was set at

(2) $$t_{n}=\frac{1}{2\nu }\ln\left(\frac{1}{1-2nd}\right)$$

for 0<*nd*<0.5. As the Laplace distribution is an even function (i.e. symmetrical around the *y* axis),

(3) $$t_{-n}=-t_{n}$$

To test the validity of using the Laplace distribution to model expected temporal delays between sEJPs and NCTs, the electrophysiological record in each experiment was shifted by a random increment relative to the fluorescence trace (at least 5 s away from the raw trace; a circular boundary condition was applied) and both the median delay and the chi-square value were recalculated. As such a shift is significantly greater that 1/ν then a shift should remove any temporal correlation. In all cases, there was no resulting significant difference between the resulting observed delays (where sEJP had been shifted) and the expected delays as predicted by the Laplace distribution (*P*>0.2, *n*=5).

Occasionally, sEJPs occurred with a high local frequency. Correlating the amplitude and timing of these sEJPs with the corresponding Ca^2+^ fluorescence was difficult due to the long time course of NCTs, such that several sEJPs were often observed to correlate with what appeared to be a single NCT. For this reason, such events were not included in the analysis.

### Statistical analysis

**Fig. 1:**
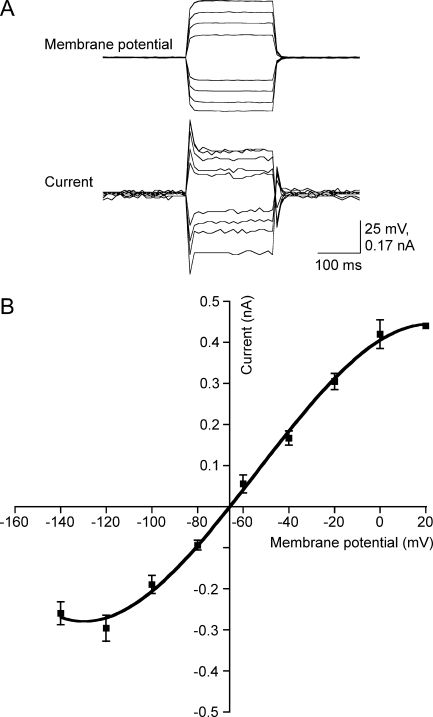
Voltage-clamp of surface SMCs. (A) Cells were voltage-clamped at various values with respect to their resting membrane potential. Data were down-sampled to 100 Hz for presentation. (B) The resulting current–voltage relationship (*n*=4) is centered around the mean resting membrane potential, which was −65.1±2 mV. Points plotted are mean±S.E.M. The fitted line is a third-order polynomial.

For other statistical tests, the normality and homogeneity of variance were tested prior to statistical analysis. Fitting of a third-order polynomial to the I:V relationship of surface SMCs (Fig. 1B) was performed using GraphPad Prism 4.0b (GraphPad Software Inc., San Diego, CA, USA). To test the variance of evoked EJP amplitudes in surface and deeper cells using the *F* test, the EJP amplitudes were normalized to the mean amplitude per cell. The term *n*, used in the presentation of statistical analyses throughout, refers to the number of animals.

## Results

### EJPs and electrical coupling

Surface SMCs were voltage-clamped to determine their input resistance, to give an indirect measure of their electrical coupling. The mean resistance was 176±18 MΩ (mean±S.E.M., range: 141–221 MΩ, *n*=4; Fig. 1).

**Fig. 2:**
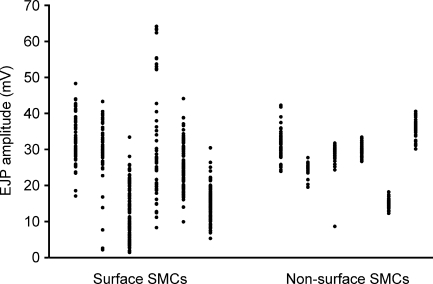
The amplitude of electrically-evoked EJPs is more variable in surface SMCs, than non-surface SMCs. Within each surface SMC, electrically-evoked EJPs are more variable in amplitude (*n*=6) than those from non-surface (i.e. deeper) SMCs (*n*=6). For more traditional amplitude frequency histograms, see Supplementary Fig. 1.

Another test of electrical coupling is the variability of EJP amplitude. Electrically-evoked EJPs were recorded from surface and non-surface SMCs (Fig. 2). Electrically-evoked EJPs from surface SMCs were more variable in amplitude (S.D.=13.4 mV; *n*=6) than those recorded from non-surface (i.e. deeper) SMCs (S.D.=8.2 mV; *n*=6) (*F* test, *F*_244,542_=17.4, *P*<0.0001).

### LTX-stimulated sEJPs and NCTs

**Fig. 3:**
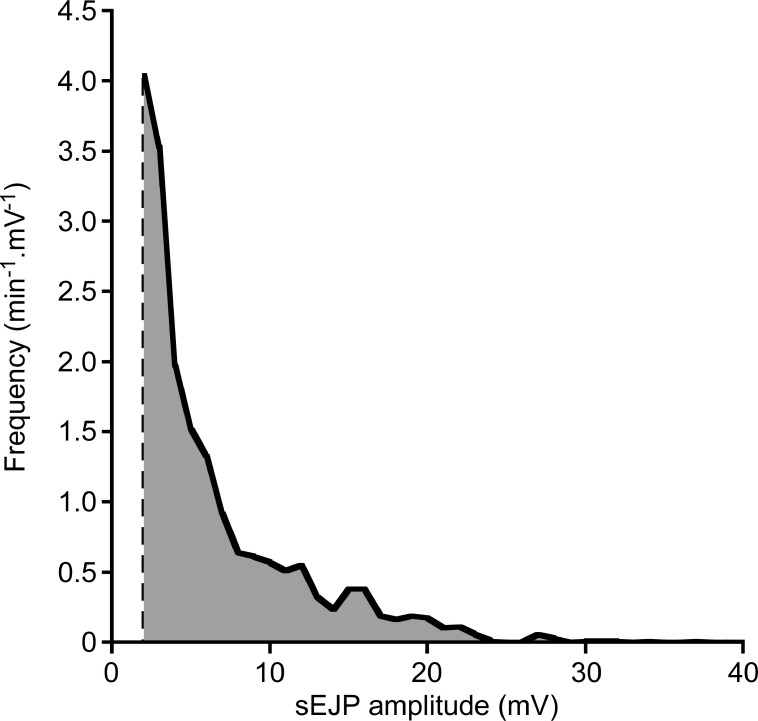
Amplitude distribution of LTX (25 pM)-stimulated sEJPs in surface SMCs (*n*=4). The threshold for detecting sEJPs, represented with the dashed line, was 2 mV.

An extract of the black widow spider venom, LTX was used to increase sEJP and NCT frequency, so that a sufficient number of events could be recorded before the Ca^2+^ indicator was photobleached. LTX (25 pM)-stimulated sEJPs in surface SMCs had a right-skewed distribution (Fig. 3), ranging from 2 mV (the threshold for detection) to 37 mV (*n*=4). Superfusion of LTX (25 pM) for 30 min, in the presence of the α_1_-adrenoceptor antagonist prazosin (100 nM), increased the frequency of spontaneous NCTs to 220±70% of controls (*P*<0.05, *n*=6, one-tailed paired *t*-test; NCT frequency measurement according to Brain et al., 2003) but did not affect their amplitudes (amplitudes 111±14% of controls, *P*=NS, *n*=6, two-tailed paired *t*-test). NCTs have previously been shown to require the activation of P2X_1_ receptors (Brain et al., 2002). After applying the P2X_1_ antagonist 4,4′,4″,4‴-[carbonylbis[imino-5,1,3-benzenetriyl bis(carbonyl-imino)]]tetrakis(benzene-1,3-disulfonic acid) (NF449) (1 μM; Tocris, UK) for 1 h, the amplitude and frequency of latrotoxin-stimulated spontaneous NCTs were reduced by 59±5% and 77±8%, respectively (amplitude: *P*<0.05; frequency: *P*<0.01; *n*=6, one-tailed paired *t*-test for both comparisons). The presence of residual NCTs following superfusion of NF449 prompted a study of the effect of NF449 on EJPs. Even after 1 h exposure to a higher concentration of NF449 (10 μM), the amplitude of EJPs was only reduced by 69±9% of controls (*P*<0.01, *n*=6, two-tailed paired *t*-test).

To determine whether latrotoxin might have a direct effect on smooth muscle membrane conductance, the resting membrane potential of surface SMCs was measured. During a control period the membrane potential was −66±6 mV (mean±S.D., *n*=4), and following superfusion of LTX, the resting membrane potential (measured in between depolarizations) was −68.6±2 mV (*n*=4).

**Fig. 4:**
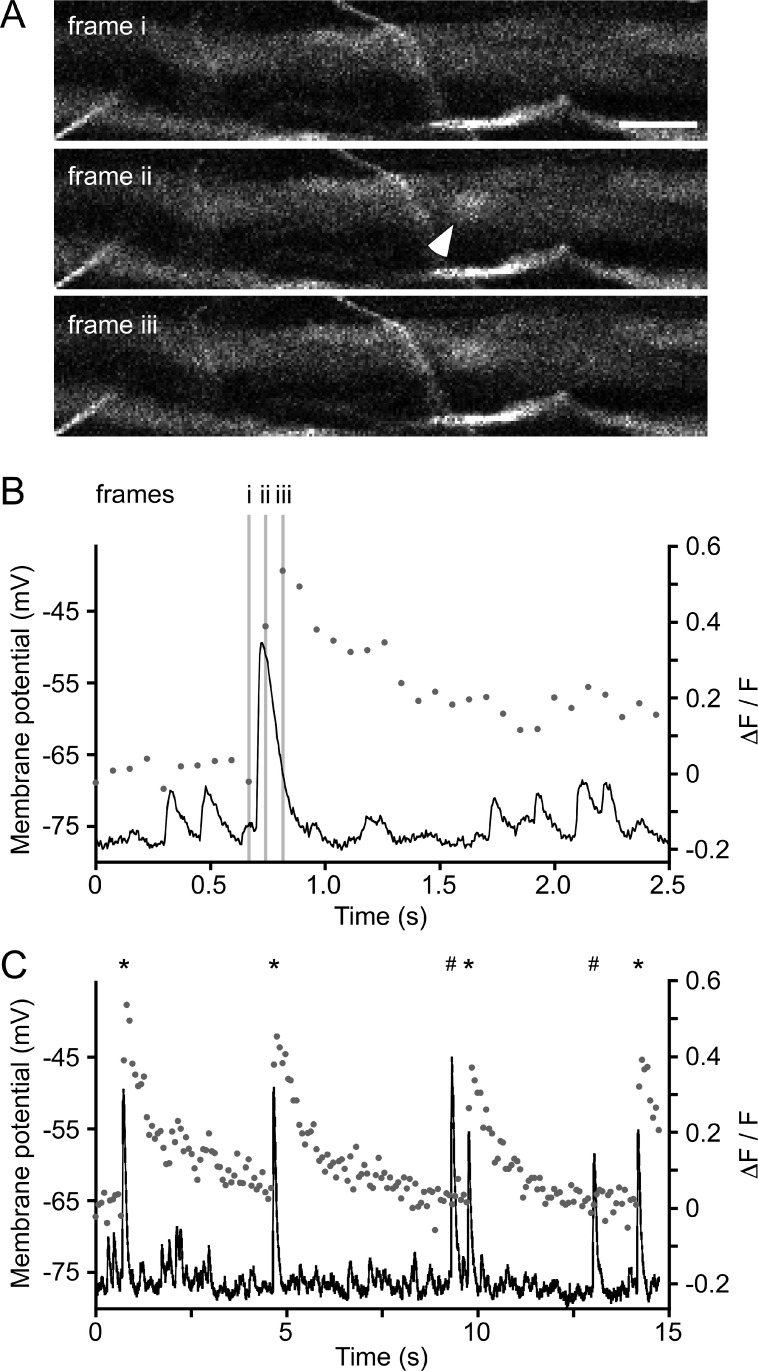
NCTs are detected within one frame of spontaneous EJPs. (A) A region of a SMC in mouse vas deferens (loaded with the Ca^2+^ indicator Oregon Green 488 BAPTA-1 AM) during an intracellular recording. Images, acquired at 13.5 Hz, are 3 frames of a 200 frame series, showing the occurrence of a LTX (25 pM)-stimulated NCT (arrow). (B) Intracellular recording of a period that includes the same three frames that compose (A), showing simultaneous recordings of membrane potential (black line) and fluorescence (gray dots). (C) Simultaneous Ca^2+^ imaging and electrophysiology showing the coincidence of NCTs and sEJPs (denoted by asterisks) during a longer recording. Not all sEJPs (for example, those events denoted by hash symbols) are coincident with an increase in fluorescence. For simplicity, asterisks and hash symbols are only shown for sEJPs greater than 10 mV in amplitude. Scale bar=10 μm.

**Fig. 5:**
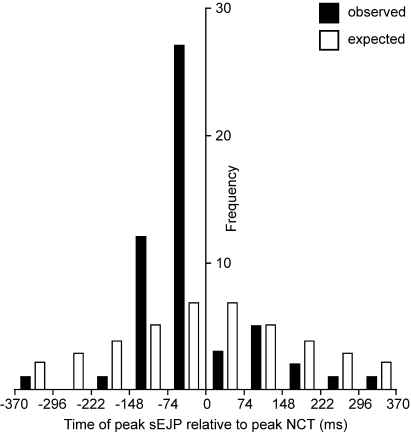
The frequency distribution of temporal delays between sEJPs and NCTs. Data are presented for a typical experiment (filled bars) and expected values (open bars), as modeled by the Laplace distribution. The boundaries between intervals are shown at one frame width (74 ms) for presentation; for analysis, they were set to ensure the expected number of events remained constant in all bins.

Simultaneous electrophysiology and confocal imaging was used to determine the relationship between the well-established method for monitoring neurotransmitter release, electrophysiology, with NCTs. Focal increases in intracellular Ca^2+^ concentration (Fig. 4A) were coincident with sEJPs (Fig. 4B, C). The time from the peak of each NCT to the nearest sEJP was measured; the median time between the peak of an NCT and the peak of the nearest sEJP ranged from −30 to −57 ms (*n*=5; Table 1). The frequency distribution of temporal relationships between sEJPs and NCTs was significantly different from an expected distribution, as modeled using the Laplace distribution (chi-square, *P*<0.05 in all experiments, *n*=5; Fig. 5, Table 1).

Many, but not all, sEJPs were associated with NCTs in the monitored region (174 NCTs with 413 sEJPs were sampled from *n*=4 experiments). However, the entire SMC could not be simultaneously viewed within one confocal optical section (mean field: 94 μm, range 81–100 μm; mean SMC length: 204±9 μm; 28 sites from *n*=6). Correcting for the average SMC length gives an estimated 0.91 NCTs per sEJP. The number of NCTs recorded (174) is not significantly different from that expected under the hypothesis that all NCTs arise from the cell recorded from (95% confidence interval, 163–217 NCTs; making no assumption about the distribution, but applying the Central Limit Theorem for large *n*), i.e. these measurements are consistent with each sEJP being coincident with a NCT occurring somewhere within the SMC.

**Fig. 6:**
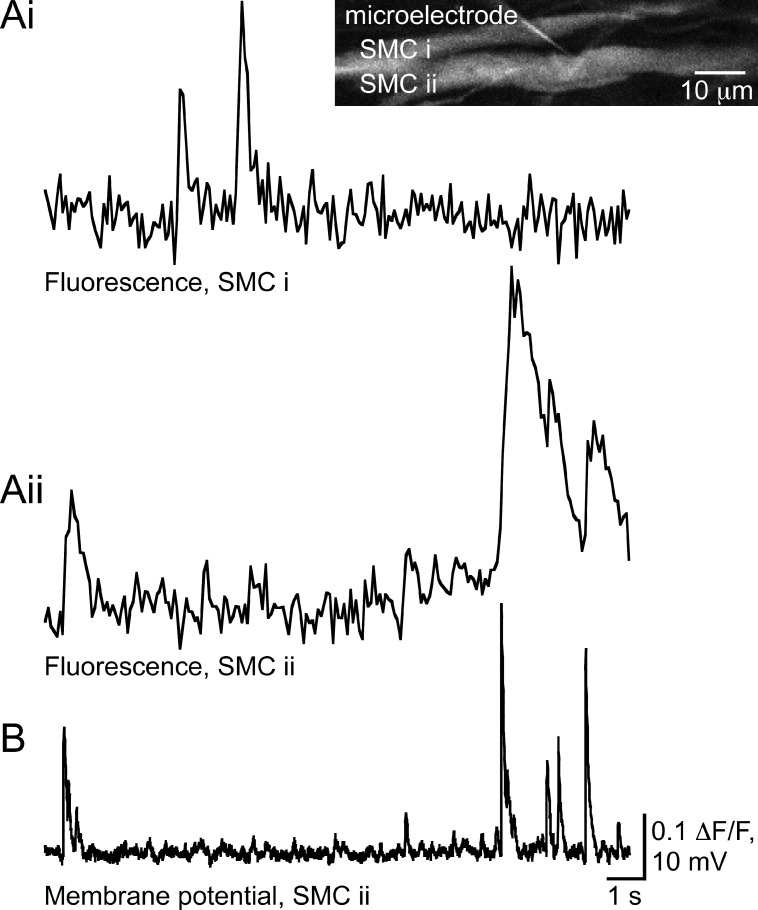
NCTs present in a single SMC do not correlate with sEJPs or NCTs recorded from an adjacent cell. (A) Ca^2+^ imaging of two adjacent SMCs (i and ii, inset). (B) Intracellular recording corresponding to SMC ii. Fluorescent images were acquired at 13.5 Hz.

If the surface SMCs are electrically-coupled, there should be a temporal correlation between sEJPs and NCTs in the adjacent cell. There was no temporal correlation between the occurrence of NCTs in adjacent SMCs (see Fig. 6 for an example). Simultaneous electrophysiology and confocal imaging revealed a good temporal correlation between sEJPs and NCTs in the impaled cell (Fig. 6Aii, B) but no correlation between NCTs in a neighboring cell (Fig. 6Ai) and either NCTs or sEJPs of the impaled cell (Fig. 6Aii, B). It was rare to observe coincident NCTs in adjacent cells. Such occurrences were often at sites where a nerve branch ran between two SMCs and have previously been suggested to arise when ATP released from a varicosity binds to P2X_1_ receptors on more than one SMC (Brain et al., 2002).

**Fig. 7:**
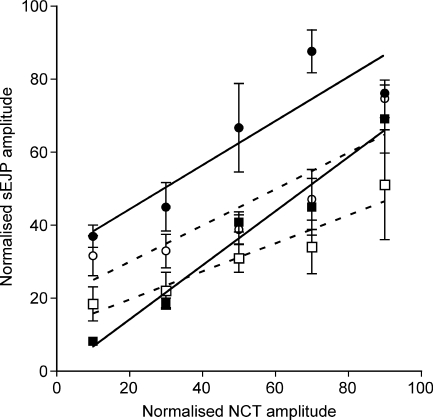
Amplitudes of sEJPs and NCTs are positively correlated (Pearson product moment correlation, *P*<0.001 for each of *n*=4 experiments, unbinned data). The amplitude correlation between sEJPs and NCTs, where both events could be simultaneously measured, suggests that they arise from the same event and that the variability in sEJP amplitude represents variability in the local action of ATP. The amplitudes were normalized to a percentage of the maximum value in each experiment. NCT amplitudes were binned and the mean amplitude of coincident sEJPs for each bin was plotted. sEJP amplitudes are mean±S.E.M. for each preparation. Lines were fitted to the binned data; the solid lines relate to the filled symbols and the dashed lines to the open symbols.

There was a correlation between the amplitudes of NCTs and sEJPs, both for large and small amplitude events (Pearson product moment correlation, ρ>0.55 and *P*<0.001 for each of *n*=4 experiments, unbinned data) (Fig. 7; for unbinned data see Supplementary Fig. 2).

## Discussion

The hypothesis that surface SMCs are poorly coupled is supported by several observations. First, input resistances of surface SMCs (176±18 MΩ, range: 141–221 MΩ) are comparable to dispersed cells (331±43 MΩ; Blakeley et al., 1989). Input impedance for syncytial smooth muscle lies in the range of 5–30 MΩ (Bennett, 1967; Holman et al., 1977; Manchanda and Venkateswarlu, 1999). Second, the amplitude of evoked EJPs in surface SMCs is shown to be more variable (S.D.=13.4 mV) than those from non-surface (i.e. deeper) SMCs (S.D.=8.2 mV). An increase of EJP variability has been previously reported in the guinea-pig vas deferens when SMCs are uncoupled with heptanol (Manchanda and Venkateswarlu, 1999). Third, the resting membrane potential (−66 mV) is more positive than that of SMCs recorded elsewhere, being similar to values recorded from dissociated cells (−45 mV; Blakeley et al., 1989).

The variable amplitude of sEJPs has been attributed to the electronic attenuation of depolarizations originating from distant release sites i.e. on neighboring cells (Tomita, 1970; Purves, 1976). Evidence for quantal neurotransmission from sympathetic nerves without the confounding effects of electrotonic attenuation comes from the work of Hirst and Neild (1980). Using short segments of guinea-pig isolated gut submucosal arterioles, they reported that the distribution of sEJPs was monophasic but highly right-skewed, particularly after deconvolving to account for recording noise (see their Fig. 3). In our experience, such a distribution is difficult to distinguish from an exponential distribution when sEJPs are counted manually, as it is difficult to identify small amplitude events close to the noise. We did not observe a similar distribution of sEJPs in the surface cells of the vas deferens, despite the fact that the expected number of close contact varicosities should be smaller (i.e. those contacting just one SMC, rather than the 100–200 varicosities in the short arteriole segments). It is possible that the signal-to-noise ratio in the present work was not sufficient to identify a peak in the amplitude-frequency histogram, but our experiments do confirm a highly skewed rather than Gaussian distribution of sEJPs. In this sense there is agreement that neurotransmitter release cannot be the sum of units of constant size. Hirst and Neild (1980) also argued that the EJP amplitude distribution could be constructed as an arbitrarily weighted sum of integral multiples of the sEJP distribution, but they themselves noted that such a construction was not possible if the raw data were deconvolved to correct for electrical noise, and they also noted that the arbitrary weighting in the sum did not yield the expected binary distribution of amplitude coefficients. These two observations imply that even in their work the EJP could not be explained as the sum of independently released neurotransmitter packets.

It is unlikely that the skewed frequency distribution of sEJP amplitudes can be explained by variable distances between varicosities and the electrode; this would result in a weak correlation or no correlation between the amplitudes of NCTs and sEJPs, rather than the strong positive correlation reported. An alternative model for neurotransmission (the modular hypothesis; Edwards et al., 1990) where neurotransmitter packets act on small clusters of readily-saturated receptors, is not supported by the findings of a broad sEJP amplitude distribution and no evidence of a multimodal EJP amplitude distribution exists. Bennett et al. (1995) similarly concluded, from their own Monte Carlo simulations, that the modular hypothesis is not tenable at this junction.

In addition to temporal coupling, NCTs and sEJPs were also correlated in amplitude throughout the amplitude range. It has been suggested that spontaneous NCTs only arise from the release of the contents of giant dense-cored vesicles (Blair et al., 2003) rather than all vesicle types. This now seems unlikely given the good temporal (and hence frequency) correlation between sEJPs and NCTs described in the present work. It is improbable that both sEJPs and NCTs reflect release from only one population of large vesicles. Others have shown that more than one basic mechanism of ATP release occurs from terminals in the mouse vas deferens by using peptide fragments of α–soluble *N*-ethylmaleimide-sensitive factor attachment protein (α-SNAP) (Bennett et al., 2001), and it will be of interest to see whether it is this SNAP-dependent pathway that generates NCTs. The present observations do not exclude the possibility of fractional release of the neurotransmitter contents of each vesicle, as suggested to occur in other innervated tissues (Ceccarelli et al., 1972; Harata et al., 2006).

A limitation of this study is that the entire SMC count could not be monitored simultaneously, so it was not possible to match each NCT with a sEJP. However, the estimate of 0.91 NCTs per sEJP (with a 95% confidence interval of 0.78–1.06) is consistent with the hypothesis that each sEJP is associated with an NCT. Such a temporal correlation is unexpected, for should SMCs be well coupled, the frequency of sEJPs should vastly exceed that of NCTs. Furthermore, NCTs in adjacent cells were not temporally coupled. These findings provide further evidence for poor electrical coupling between surface SMCs.

Within the limits of our recording system (i.e. 13.5 Hz confocal microscopy acquisition rate) the present study demonstrates that focal increases in intracellular Ca^2+^ are tightly temporally coupled to the electrical sign of spontaneous neurotransmitter release in this tissue; i.e. the sEJP. Hence, the present work validates the use of NCTs to monitor neurotransmitter release, with the proviso that some NCTs may arise from the superposition of bursts of spontaneously-released neurotransmitter packets. The rate at which images were sampled limits the temporal resolution with which sEJP recordings can be compared with NCTs. Using line scanning confocal microscopy it has been reported that electrically-evoked NCTs can be detected 6 ms after stimulation (Brain et al., 2002), suggesting that the real delay between the sEJP and NCT is much briefer than the range of values, −30 to −57 ms, reported here (*n*=5; Table 1). NCTs were not generated by the activation of α_1_-adrenoceptors because they were observed in the presence of the α_1_-adrenoceptor antagonist prazosin (100 nM) as previously described (Brain et al., 2002). The observation that NF449 greatly reduced NCT amplitude and frequency (presumably as the postjunctional response fell below the detection threshold), is consistent with previous reports that NCTs are abolished or greatly reduced by α,β-methylene ATP (Brain et al., 2002, 2003), showing that they reflect the action of ATP at P2X_1_ receptors. The observation that NCTs remain in the presence of the P2X_1_ antagonist NF449 (1 μM) is consistent with the observation that the desensitizing agonist α,β-methylene ATP does not abolish all excitatory junctional potentials (or currents; Allcorn et al., 1986; Liang et al. 2000) or NCTs (Brain et al., 2003), and more importantly that NF449 did not abolish EJPs after 1 h of exposure (the present study). Given that EJPs do not occur in P2X_1_ knockout mice (Mulryan et al., 2000) it may be that there is a proportion of sites that are either insensitive to these drugs or inaccessible within the incubation time used.

The time course of NCT recovery (1.8 s; 174 NCTs, *n*=4) was significantly slower than previously reported, when the fluorescent signal was measured over a smaller area (280 ms; Brain et al., 2002). This dependence of the time course on the area in which the signal is measured is consistent with most models of near-membrane Ca^2+^ kinetics in SMCs (Kargacin and Fay, 1991). Also, the duration of the NCT is consistent with observations from the urinary bladder (Heppner et al., 2005). Occasional bursts of sEJPs led to a fused local Ca^2+^ transient that could easily be interpreted as one NCT. This latter finding suggests that the amplitude distribution of spontaneous NCTs cannot be relied upon to reflect the response of single packets of neurotransmitter unless simultaneous electrophysiological recording is used or the temporal resolution of the optical detection system is improved.

The amplitude of NCTs at a given junction can vary by more than ninefold (Brain et al., 2002) but given that a significant proportion of the amplitude is due to (potentially highly variable) amplification by Ca^2+^-induced Ca^2+^ release (Brain et al., 2003), it is not possible to attribute variation in the NCT amplitude solely to variability in the neurotransmitter packet size, or even to variation in the number of P2X receptors opened. For this reason, measuring the amplitude distribution of sEJPs is a more direct measure of variation in neurotransmitter packet size than the measurement of NCT amplitudes.

### Latrotoxin as a tool to study spontaneous neurotransmitter release

Both crude black widow spider venom and purified LTX have been extensively used to study molecular neurotransmission in vertebrates (e.g. Ceccarelli and Hurlbut, 1980; Hurlbut et al., 1990; Auger and Marty, 1997) with the established LTX concentration used often in the low nanomolar range (1–2 nM; Hurlbut et al., 1994; Davletov et al., 1998; Silva et al., 2005). Yet low picomolar concentrations (25 pM, the present study; 130 pM, McMahon et al., 1990) have been shown to increase marginally the rate of neurotransmitter release. In brain synaptosomes (Nicholls et al., 1982) LTX increased the rate of neurotransmitter release even in the presence of the Na^+^ channel blocker tetrodotoxin, implying that its action is independent of sodium-dependent nerve action potentials. Despite these very low concentrations it is possible that LTX-stimulated sEJPs do not have the same properties as true sEJPs. While there was no effect of this low concentration of LTX on the resting membrane potential, and no direct SMC effects have been reported at this concentration, it is possible that latrotoxin caused a small conductance change which was well balanced by other conductances. This possibility was not investigated.

### Variability in the electrical properties of surface SMCs

Despite being depolarized to greater than 0 mV during voltage clamp, only two of four cells exhibited active currents. This variability in the cells’ responses to depolarizing steps contrasts to the opening of both inward and outward currents in response to depolarizing voltage steps reported by Blakeley and colleagues (1989) but is consistent with the observations of Holman and colleagues 1977; compare their Fig. 3A and 3B). Variability in the electrical properties of surface SMCs is also demonstrated in recorded resting membrane potentials: −40 to −80 mV (Blakeley et al., 1989) and −60 to −72 mV (the present study). A likely explanation for the variance in resting membrane potential, and responses to depolarizing voltage steps of surface SMCs, is a varying degree of electrical coupling. Moreover, ‘coupled’ and ‘uncoupled’ represent two ends of a spectrum; surface cells are somewhere toward the ‘electrically-uncoupled’ extreme.

Simultaneous electrophysiological recording and confocal microscopy now reveals that NCTs are coupled in timing and in amplitude with sEJPs, implying that NCTs provide a sensitive, high-resolution technique to monitor spontaneous neurotransmitter release. The observation of a wide and skewed amplitude distribution of sEJPs in cells that are very poorly coupled weakens the argument for neurotransmission of a uniform packet size at autonomic neuroeffector junctions. It is likely that each packet corresponds to the release of the neurotransmitter contents of one vesicle, but whether the variability in the postjunctional response to neurotransmitter packets results from variable neurotransmitter release, or from variable postjunctional receptor responses to consistent neurotransmitter release, remains to be established.

## Figures and Tables

**Table 1:** The temporal relationship between NCTs and sEJPs (*n*=5)

Characteristic	i	ii	iii	iv	v
No. of NCTs	36	36	24	12	54
Median peak NCT vs. peak sEJP (ms)	−57	−46	−42	−37	−30
25, 75% Quartiles (ms)	−122, 77	−91, 13	−74, 10	−71, 24	−74, 3
Chi-square test, *P*	<0.001	<0.001	<0.0001	<0.05	<0.0001

## Footnotes

Appendix Supplementary data associated with this article can be found, in the online version, at doi: 10.1016/j.neuroscience.2006.11.054.
