# Supplementary material for: The origin of the skewed amplitude distribution of spontaneous excitatory junction potentials in poorly coupled smooth muscle cells
Source: Neuroscience. 2007 Mar 2;145(1-5):153–61. doi: 10.1016/j.neuroscience.2006.11.054 (PMC2543106; doi:10.1016/j.neuroscience.2006.11.054)
Supplement: Supplemental Fig. 1 [file mmc1.pdf]

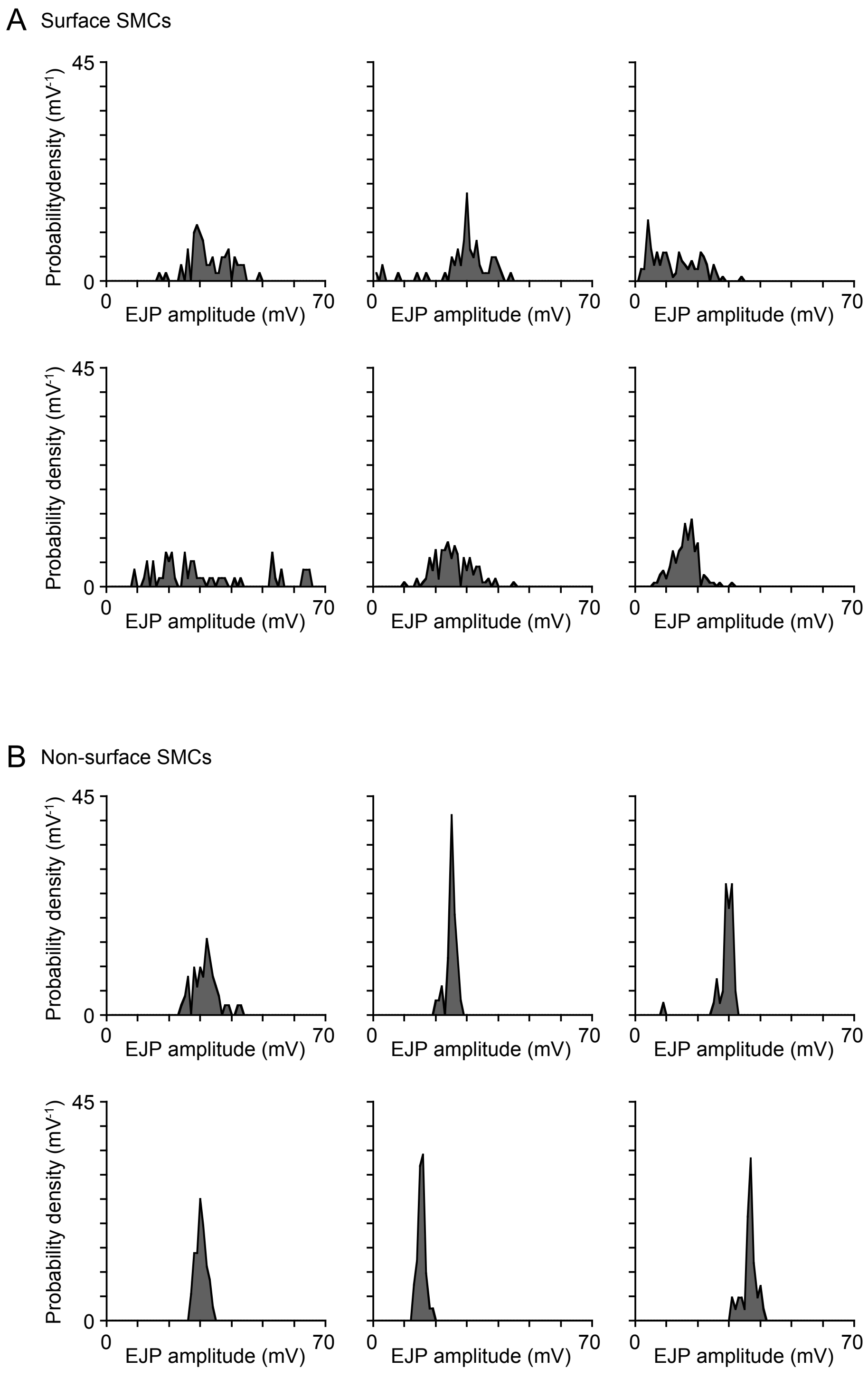

Supplementary Figure 1. Amplitude distributions of EJP amplitudes of surface (A) and non-surface (B) SMCs. Data correspond to those used in Figure 2.
