# Supplementary material for: The origin of the skewed amplitude distribution of spontaneous excitatory junction potentials in poorly coupled smooth muscle cells
Source: Neuroscience. 2007 Mar 2;145(1-5):153–61. doi: 10.1016/j.neuroscience.2006.11.054 (PMC2543106; doi:10.1016/j.neuroscience.2006.11.054)
Supplement: Supplemental Fig. 2 [file mmc2.pdf]

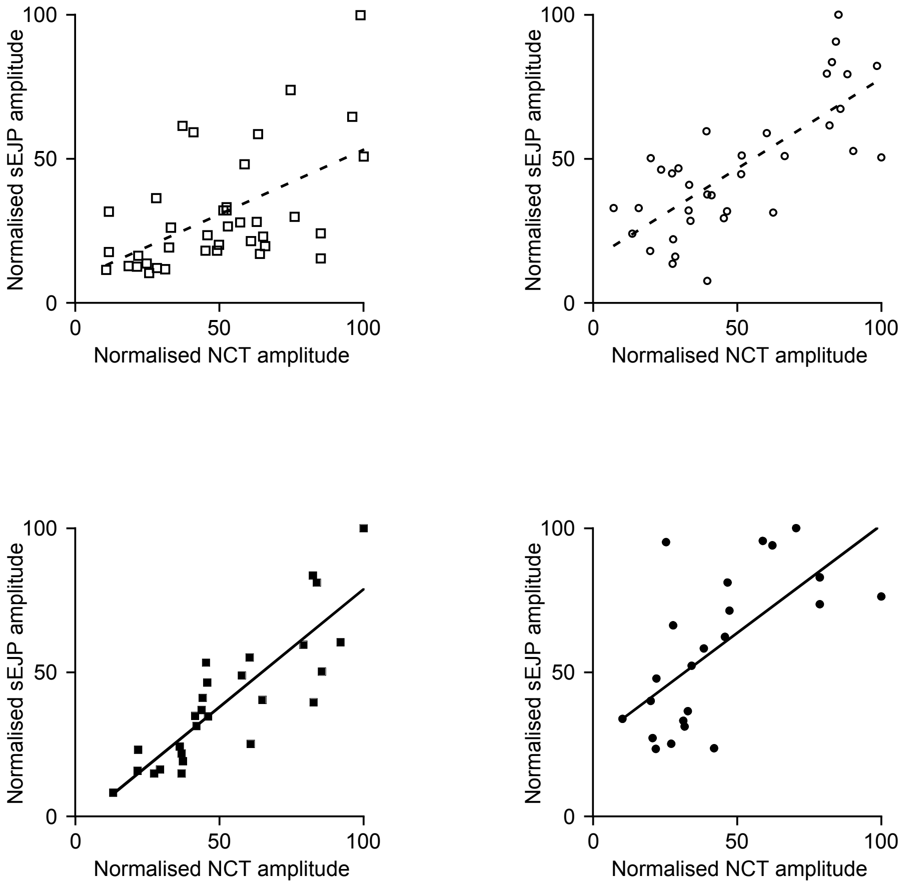

Supplementary Figure 2. The correlation in amplitudes of NCTs and sEJPs (*n* = 4) where both events could be simultaneously measured. Data are unbinned. Symbols, lines and data sets correspond to those used in Figure 7.
